# Persistent barriers to the use of maternal, newborn and child health services in Garissa sub-county, Kenya: a qualitative study

**DOI:** 10.1186/s12884-020-02955-3

**Published:** 2020-05-07

**Authors:** Isaac Kisiangani, Mohamed Elmi, Pauline Bakibinga, Shukri F. Mohamed, Lyagamula Kisia, Peter M. Kibe, Peter Otieno, Naïm Afeich, Amina Abdullahi Nyaga, Ngugi Njoroge, Rumana Noor, Abdhalah Kasiira Ziraba

**Affiliations:** 1 Health and Systems for Health Research Unit, African Population and Health Research Center, P.O. Box 10787, Nairobi, 00100 Kenya; 2 Preventive Health Care, P.O. Box 639, Wajir, 70200 Kenya; 3 Department of Epidemiology, Biostatistics and Occupational Health, McGill University, 1020 Pine Avenue West, Montreal, QC H3A 1A2 Canada; 4 Sisters Maternity Home, P.O. Box 545, Garissa, 70100 Kenya

**Keywords:** Barriers, MNCH, Sociocultural factors, Garissa, Kenya

## Abstract

**Background:**

North Eastern Kenya has persistently had poor maternal, new-born and child health (MNCH) indicators. Barriers to access and utilisation of MNCH services are structural, individual and community-level factors rooted in sociocultural norms. A package of interventions was designed and implemented in Garissa sub-County aimed at creating demand for services. Community Health Volunteers (CHVs) were trained to generate demand for and facilitate access to MNCH care in communities, while health care providers were trained on providing culturally acceptable and sensitive services. Minor structural improvements were made in the control areas of two facilities to absorb the demand created. Community leaders and other social actors were engaged as influencers for demand creation as well as to hold service providers accountable. This qualitative research was part of a larger mixed methods study and only the qualitative results are presented. In this paper, we explore the barriers to health care seeking that were deemed persistent by the end of the intervention period following a similar assessment at baseline.

**Methods:**

An exploratory qualitative research design with participatory approach was undertaken as part of an impact evaluation of an innovation project in three sites (two interventions and one control). Semi-structured interviews were conducted with women who had given birth during the intervention period. Focus group discussions were conducted among the wider community members and key informant interviews among healthcare managers and other stakeholders. Participants were purposively selected. Data were analysed using content analysis by reading through transcripts. Interview data from different sources on a single event were triangulated to increase the internal validity and analysis of multiple cases strengthened external validity.

**Results:**

Three themes were pre-established: 1) barriers and solutions to MNCH use at the community and health system level; 2) perceptions about women delivering in health facilities and 3) community/social norms on using health facilities. Generally, participants reported satisfaction with services offered in the intervention health facilities and many indicated that they would use the services again. There were notable differences between the intervention and control site in attitudes towards use of services (skilled birth attendance, postnatal care). Despite the apparent improvements, there still exist barriers to MNCH services use. Persistent barriers identified were gender of service provider, insecurity, poverty, lack of transport, distance from health facilities, lack of information, absence of staff especially at night-time and quality of maternity care.

**Conclusion:**

Attitudes towards MNCH services are generally positive, however some barriers still hinder utilization. The County health department and community leaders need to sustain the momentum gained by ensuring that service access and quality challenges are continually addressed.

## Background

Global efforts to improve maternal, new-born and child health (MNCH) have been reconfirmed through the Sustainable Development Goals (SDGs) agenda that challenges countries to further consolidate the efforts to reduce the global burden of maternal, new-born and child mortality [1]. In the 25 years before SDGs, global trends showed improvements in MNCH indicators with many sub-Saharan Africa (SSA) countries showing a drop in maternal deaths and an increasing proportion of skilled birth attendance [2]. Despite the global efforts and progress, maternal deaths are still high in SSA and southern Asia, both these regions accounted for about 86% of such deaths globally [2]. In Kenya, the maternal mortality ratio is still high at 520 maternal deaths per 100,000 live births while child mortality is estimated to be 52 deaths per 1000 live births [3]. The modest decline has been attributed to various prevailing factors including infectious diseases such as a HIV/AIDS, high blood pressure during pregnancy, complications from delivery and unsafe abortion, limited access and utilization of health facility delivery services especially in some sub-populations [2, 4].

The determinants for low MNCH utilization are contextualized factors from both the user (demand-side) and the health system factors (supply-side) [4, 5]. The user factors often include challenges such as distance to the health facility, cultural beliefs and practices, economic factors and many other factors operating within the community and household of users [5]. Health system factors could range from capacity of health staff, attitude of care providers, infrastructure and other forms of interactions with a health care system by users [6].

The Kenyan government’s efforts in addressing cost barriers, by making public health facility deliveries free, have not yet resulted in increased skilled birth attendance across the country [7, 8]. For instance, in 2014 only 62% of the births in Kenya were conducted with the help of a skilled health care worker [3]. Furthermore, other MNCH services such as focused antenatal care (FANC), timely childhood immunization and postnatal care (PNC) have also remained low nationally [8]. Despite the Kenyan government efforts in improving MNCH, Counties in the North Eastern part of Kenya in particular Garissa, have the poorest MNCH indicators and have been labelled as high burden areas [3, 8].

Garissa is part of the larger North Eastern part of Kenya which is a semi-arid area with majority of the local residents being nomads. The majority of the local residents are of Somali ethnic group extraction and are predominantly Muslim. Compared to many other parts of Kenya, North Eastern Kenya is less endowed with social amenities such as schools, safe water, health facilities, and paved roads [9]. Culturally, girls are married off at a relatively young age in line and female genital mutilation is common practice in this region [3, 10, 11]. Due to the geographical area, ethic proximity and refugee influx from Somalia, North Eastern Kenya continues to face insecurity [12]. This precarious environment limits humanitarian access and service delivery with the education and health sectors most affected [13].

Most of the barriers to healthcare access are rooted in the local cultural norms and practices [14–17]. Medicalization of the birthing process that ignores social and traditional norms surrounding this family event is one of the reasons for home deliveries. To increase skilled delivery in Garissa, it was critical to conduct strategic social mobilization, while addressing key inherent health system barriers through the provision of culturally acceptable and sensitive services (CASS) at healthcare facilities. Traditional institutions, such as the clan (*Reer*) and the Somali customary law (*Heer*) are dominant and important institutions in Garissa County and North Eastern Kenya. These traditional social systems are strong, have a sense of local ownership and generate healthy competition among the local people. They have been used in solving disagreements and conflict, allocating resources and agreeing on contributions to important matters in the community but have not been well utilized by the health sector [18–20]. The need to explore effective strategies rooted in existing healthcare and socio-cultural matrices in order to promote institutional deliveries was at the heart of this project. In this regard, the baseline survey helped us understand the situation better and informed tailoring of proposed interventions to fit the context of the community. The pre-intervention study cited male nurses and doctors attending to women in labour, living far from the health facility, lack of a proper and reliable means of transport, poor attitude of healthcare providers, limited availability of healthcare workers, equipment and supplies and lack of awareness and information on the importance of skilled birth attendance as barriers in using health facility delivery [21].

This paper is part of an impact evaluation of an innovation package aimed at improving utilization of MNCH services. The intervention package was developed through social mapping of community resources in terms of major clans, physical infrastructure, religious, health, social and political leadership, health facilities and other key social services in the area. This was achieved through social dialogue to understand their MNCH status, challenges faced and devise solutions to improve their MNCH status. In brief, the intervention involved mobilization and training of CHVs who provided health education, referrals, data collection and monthly reporting, creation of an influencer’s forum to hold the community and service providers accountable, male champions to promote MNCH service use at community level using different forums. On the supply-side, service providers in the two facilities in the intervention site were trained on culturally acceptable and sensitive service as collectively defined by the stakeholders in inception stage of the project; minor infrastructural upgrade as well as creation and supporting the functioning of two maternal perinatal and death surveillance response (MPDSR) committees. This paper aimed at exploring the persistent barriers to the utilization of MNCH services among the Somali community in Garissa Sub County following 18 months (August 2017 to April 2019) of implementing a multi-pronged package of interventions. This qualitative research was part of a larger mixed methods study on effect of cultural innovation on MNCH service utilization. In this paper, only the qualitative results are presented.

### Conceptual framework

**Fig. 1:**
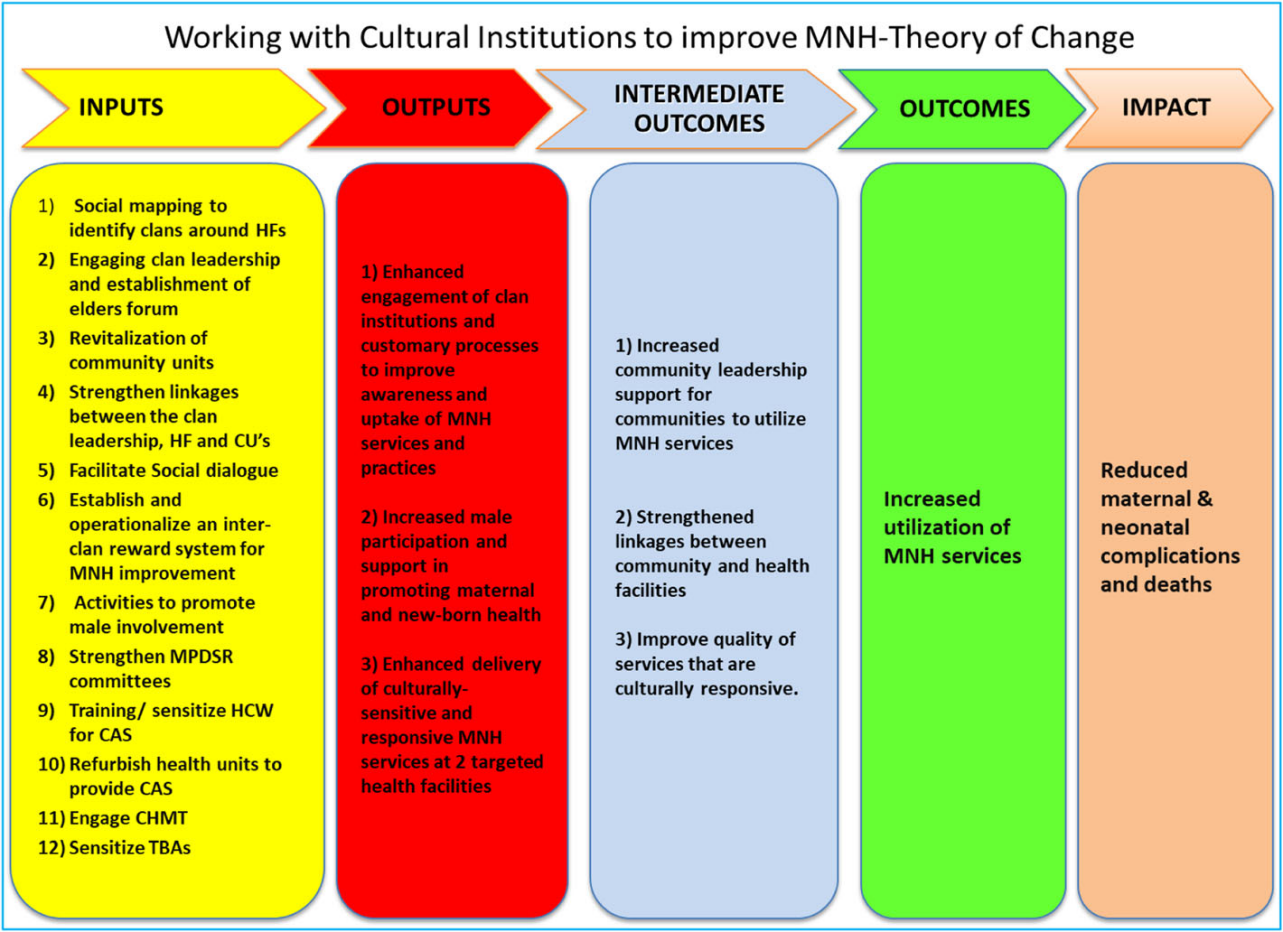
Project theory of change

The project interventions were designed to address barriers to accessing and utilizing MNCH services in Garissa sub-County. The project explored effective strategies rooted in existing healthcare and social matrices to increase utilization MNCH services [22]. A theory of change was developed by the research team to guide evaluation design and underlying mechanism through which the project might improve MNCH outcomes: 1. Increase demand for MNCH services; and 2. Improve birthing experience as shown in Fig. 1.

## Methods

### Study site

The study was conducted in three locations within Garissa sub-County. Iftin and Township were selected as intervention sites while Madina was the control site. In each of the three locations, there are community units (CUs) overseen and served by Community Health Volunteers (CHVs) who in turn report to the respective designated health facilities. Iftin location is served by Iftin sub-County Hospital, Township area is served by Sisters Maternity Home (SIMAHO) and Madina area is served by Madina Health Centre. The two intervention sites received the intervention package and the control site received the usual standard of care.

### Study design

The study utilized an exploratory qualitative design with participatory approach using focus group discussions (FGDs) and Key informant interviews (KIIs) as part of impact evaluation of the project. Purposive sampling procedures were adopted to identify and conveniently select a sample of opinion leaders (*n* = 15), women of reproductive age -who had childbirth experiences during intervention period (*n* = 53) and married men (*n* = 57) to provide data in different qualitative interviews. Health managers were purposively selected to submit further insights into expectant mother-ANC provider relationships and uptake of medical services. All the participants were approached by either email or trained research assistants to participate in the interviews. In total, 150 participants were approached and 25 refused to participate citing “lack of confidentiality” and “busy schedules”. A summary of all participants can be found in Table 1.

**Table 1:** Distribution of participants

Data collection type	Participants	Sites	No. of interviews
Focus Group Discussions (FGDs)	Married women (15–29 & 30+ years)	Township, Madina, Iftin	6
	Married men (15–29 & 30+ years)	Township, Madina, Iftin	6
	CHVs	Iftin	1
	Male champions	Township	1
Key Informant Interviews (KIIs)	Members of County Assembly (MCA)	n/a	2
	Imams/ Sheikh	Township, Madina, Iftin	3
	Clan leaders	Township, Madina, Iftin	4
	TBA	Iftin	1
	Health Managers	SIMAHO, Madina, Iftin health facilities	3
	NGOP	n/a	2
Total			29

### Research team

Some of the interviews were conducted by two of four authors (IK, RN, AAN & AKZ) who are researchers with a professional interest in MNCH. All have a health background and had experience with in depth interviews. Prior to the interviews, none of the participants were known to the interviewers, either personally or professionally.

### Research instruments

Initial interview guides were based on questions the researchers had identified after studying literature and then adjusted throughout the study as new themes arose. An interview guide containing structured and unstructured questions were applied to health professionals and opinion leaders. A similar semi-structured discussion guide was used for FGDs with community members to elicit in-depth community perspectives of the project interventions, the causes of poor MNCH outcomes, sociocultural beliefs and practices impacting the use of maternal and newborn health services and barriers to healthcare utilization.

### Data collection

Data were collected between March 2019 and April 2019 and all participants gave verbal consent for their quotes to be used. All the interviews took place in a location chosen by the participants, which was most often either in their home or place of work and rarely in a public place like health facilities. All interviews were done in private. The interviews were allowed to take a spontaneous course and lasted between 45 and 60 min.

A total of 14 FGDs and 15 KIIs were conducted. Table 1 represents the type of interviews conducted and distribution of study participants using the different study methods. In each study site, a tested interview guide was used to obtain information primarily on: (i) views on women giving birth in health facilities; (ii) barriers in accessing and using MNCH services at the community and health facility level, and (iii) possible quality improvement at health facilities. All field interviewers were trained on the study rationale, the objectives, the study approach and the data collection procedures. Interviews were conducted in Somali by trained Somali native research assistants. A note taker worked alongside with the interviewer taking notes. In both KIIs and FGDs interviews, the research assistant let the participant respond and then probed where necessary to obtain more information before proceeding to the next topics.

FGD interviews included women of reproductive age (who had ever given birth over the intervention period) and men (whose wives had delivered during the intervention period) at the community level, key informants included community leaders (religious, clan leaders), NGO personnel (NGOP), healthcare providers/managers (HM), traditional birth attendants (TBAs), political leaders MCA) and community health volunteers (CHVs). Two FGDs were conducted in each of the three study sites among married women aged 15–29 and 30 and above separately. Similarly, two FGDs were conducted with married men aged 15–29 years and the other with older men (30 years and above) in each site. We also conducted FGD among male champions. Each focus group discussion had 6–10 participants.

### Data management and analysis

Audio files were transcribed verbatim into English by native Somali speakers. The transcription was supplemented by field notes taken by note taker. The transcripts were marked according to the area where the interviews took place, date of discussion, type of group in terms of gender and role in the community. The transcripts were analyzed using content analysis, by reading through the transcripts to code important information. Two members of the research team reviewed and coded the transcripts. After coding the transcripts, the two members of the research team identified patterns from the coded data and made connections to recurrent themes and pre-established themes from the quantitative survey -such as views on health facility delivery, reasons for home delivery. Outcomes were compared for consensus. A discussion was held in case of mismatch and an agreement arrived upon to assign appropriate codes. Due to the fact that the target population share the same culture and views, data saturation occurred at the analysis stage. Themes were re-appearing in most transcripts, relaying the same information.

Data were coded using QSR International’s NVivo 12 software to identify primary and meta-codes and major themes. The themes were identified with attention to contradictions and diversity of experiences, perception and attitudes across different interviewees. The coding frame agreed by both researchers was used to systematically assign the data to the thematic categories. Coding was undertaken by a single researcher. The interpretation was undertaken by at least two members of the research team to ensure objectivity and consistency of coded information. Data from different participant groups were analyzed separately and then compared for areas of convergence and divergence. Interview data from different sources (women, their partners, opinion leaders and their health care professionals) on a single event were triangulated to increase the internal validity of this study [23]. Analyzing multiple cases strengthened external validity. The following themes were pre-established: barriers and solutions to MNCH use at the community and health system level; perceptions about women delivering in health facilities and community/social norms on using health facilities.

## Results

Interviews were conducted with 125 study participants, 110 were from FGDs and 15 were from key informant interviews (Table 1). The age of the mothers ranged from 20 to 49 years with a majority being below 35 years old. Most mothers were not educated or had limited education. Maternal characteristics of the mothers are illustrated in Table 2. Barriers to access and utilisation of MNCH services, as reported by service users, community leaders and service providers, are many and some are deeply entrenched. They have been categorized into user’s (demand-side), structural and service-side barriers. To elaborate on the findings, anonymous quotations were used bearing the participants’ position in the community and the type of interview.

**Table 2:** Maternal characteristics

Maternal characteristics (*N* = 53)	n
Age range (years)	
20–25	17
>25–30	12
>30–35	10
>35–40	6
>40–45	4
>45–49	4
Parity	
1	14
2	15
3	7
>4	16
Marital status	
Married	53
Not Married	0
Education	
No Education	20
Primary	13
Secondary	16
Tertiary	4

### User’s barriers

Barriers identified from the users’ perspective include gender of service provider, financial barriers and level of awareness.

#### Gender of service provider

Participants’ view indicate the gender of the service provider was an important barrier in accessing MNCH services in this community. The study participants noted that female healthcare professionals were preferred by women in this community due to cultural beliefs while others also noted religious obligations.

This has been a recurrent theme throughout the study period. Both men and women continue to express disapproval of expectant women being attended to by male service providers. Men seem to believe that women are uncomfortable with male attendants because of the cultural and religious norms. This also came out strongly during the pre-intervention assessment. Religious leaders asserted that the notion is rooted in the Islamic faith while others posit that it is a Somali cultural norm that needs to be observed.

*“As Muslims and Somalis, our women are shy such they don’t want their private parts to be seen by a male nurse/doctor. This has forced many mothers to give birth at home rather than being assisted by male attendant during delivery. I propose more female birth attendants should be brought so that women are able to deliver in the hospital.”****KII -clan leader 1****.*

Though males tied gender preference to religion and culture, women expressed that it was an issue of personal preference. The discomfort of being attended to by a male was so strong that most preferred to deliver at home than at health facilities.

*“There are women who will not be touched by male nurses but some have to be attended to by male nurses. The reason they give is that it is breach of their privacy when male nurses put their hands in women vaginas and also seeing them naked.”****FGD women (15-29 years)-Iftin****.*

Service providers and mangers are generally aware about this concern and as a response, the County has started addressing through affirmative action in the recruitment process, whereby female health workers intended for maternity services are given preference. This preference for a specific gender to offer services to pregnant women and postpartum mothers has resulted in most of the female healthcare workers being overworked.

“*Some employees overwork especially the female staffs because the clients want to be attend to by female staff especially in the maternity and immunization. Our clients prefer female staff so our female nurses deliver services to them.”****KII-HM 2****.*

While preference for female health workers for maternity services is common, some community members have no problem with women being catered to by male health workers. They assert that the Islamic faith teachings do not prohibit male healthcare workers from providing maternity services. It is argued that the teachings are clear on distinguishing between observing a woman’s nakedness while providing health services verses observing a woman’s nakedness for pleasure. The former is permitted.

*“We encourage mothers in the delivery room that it doesn’t matter whether it’s a male or female nurse who attends to them, they are all educated and can do the job. We have many female nurses now courtesy of this project”****KII-TBA***

### Financial barriers

Poverty is rampant, and for many a choice between seeking health and meeting basic needs has to be made. This is difficult in a situation where income generating activities have been affected by the changing environmental conditions that no longer support the community’s main economic activity. Moreover, these activities are dominated by males who dictate how finances are used. Hence, in some cases women do not seek care or are unable to pay for healthcare and associated health costs like medicines.

*“A majority of the population are poor as more than 50% of the population are pastoralists in and due to climatic change and the effects of the droughts, livestock keeping is no longer a reliable source of livelihood. So financial resources are major challenge in terms of going to hospital because hospitals are not for free, even if you go to a government institution you cannot afford to buy the drugs”****KII-NGOP 2.***

*“…. people here are not well off and cannot afford the means to general hospital or money to pay for their scans. One time there was a situation of a mother who was in labour and because of her situation had to give birth at home and we had to help her”****FGD CHVs-Iftin***

While the current national political leadership has declared maternity services as being free, it is always the case that there are other non-health related costs that women have to consider when seeking care. These may include transport and food.

*“Yes, finances are a big challenge, for instance when a mother gives birth, both the mother and the new born child need a special care like special diet and medicine in order to stabilize their situation, or sometimes the facilities may be very far and they cannot afford to facilitate their transport.”****KII-NGOP 1****.*

#### Level of awareness

Though the level of awareness on the importance of MNCH increased among community members, change agents and key policy makers noted that there was still a gap in information on key health issues like prevention, health risks, and knowledge of places to access care. This has a bearing on health care seeking behaviour. Misinformation as a result of relying on uninformed sources or from sources that deliberately misinform the public was also an area of concern.

*“People in the community are not informed on the importance of the health services offered in the hospital. They are ignorant on the importance of using MNCH services and should be informed. For example, my niece did not go to the hospital for up to 8 months of her pregnancy and one-day morning she collapsed. We found out she had hypertension and needed caesarean section. If she looked after herself and attended clinic checkups, this would have been prevented”****FGD Male champions***

There are proposals to increase MNCH awareness in the community by educating them on the importance of utilizing MNCH services. By engaging CHVs, awareness creation has picked pace over the years as they carryout home visits to offer health education and are also referral agents.

*“There is need for a lot of partnerships between the community and other stakeholders to create awareness and make them understand the need of using MNCH services…ultimately we need to do a lot of sensitizations on the importance of getting services provided by qualified personnel in their respective areas”****KII HM 2***

*“There are community health volunteers who we are in contact with and help us get this information. They live among us and we go to them for any assistance, we go to them and inform them our issues and they refer us to the General hospital, SIMAHO or GK prison health facilities.”****FGD Women (30+ years)-SIMAHO****.*

### Structural barriers

There were only two types of structural barriers identified by study participants and were distance from health facilities and insecurity.

#### Distance to health facilities

While the study was carried out in a majorly peri-urban area, lack of transport was reported as a key issue both at pre and post intervention evaluation. This was problematic specifically in emergency cases where mothers required timely care. Unfortunately, they lack access to ambulance services thus opting to give birth at home or in local low-level health facilities. Women can unexpectedly develop complications during labour that may require a higher level of professional skill and amenities such as a surgical theatre. In the absence of ambulance services, such emergency cases may end up with serious adverse outcomes including death. This also meant that a lot of the burden to help women during emergencies fell on CHVs who by definition are volunteers and don’t earn an income as a result of their work with the community.

*“The mothers may go into labour at night and they cannot get the means of transport so they opt to deliver at home. It would be great if we had an ambulance in every community to help the women in their transportation”****FGD women (15-29 years)-Iftin***

Limited transportation options meant that a majority of mothers rely on public transportation or walk to health facilities. This becomes especially difficult for pregnant women in their third trimesters. Moreover, due to their other roles and responsibilities, women would rather stay at home than spend time travelling to the clinics.

“*Coming back for appointments is a challenge for mothers due to lack of means of transport, and distance from the hospital. The mother takes care of the other children and the clinics are about 4 kilometres away”****FGD women (15-29 years)-Iftin***

The transport challenge was also emphasized by local health stakeholders and service providers who pointed out that lack of transportation was a problem especially in nomadic communities that live outside the urban neighbourhood.

*“Most of this community are pastoralists, who move from one place to another, when they move they move very far from facilities and then accessing the facilities will be very difficult for them. It makes them walk a long distance or sometimes they may not have the means of transport”****KII-NGOP 1****.*

#### Insecurity

Garissa and other Counties in the region have experienced significant insecurity. Insecurity came out strongly as a major barrier to accessing and utilizing MNCH services, especially during delivery. This was mentioned to have an effect on the provision of health services particularly affecting utilization of healthcare and availability of health workers to tend to women. This has limited the operating hours in some facilities as healthcare workers are afraid to operate at night. This means reduced service availability at night. Hence, those in need cannot access care.

*“Because of lack of security, at night there are no doctors. I came here on Sunday night and there was no nurse. The watchman only. Then had to go to General hospital”****FGD women (15-29 years)-SIMAHO.***

For those who can access care, they have to leave the facilities before the recommended 24 h for observation. It has also affected movement of ambulances at night to offer emergency transport services.

*“Main one is insecurity. It affects us because no ambulance can go and fetch patients at night. They say it is insecure and what is worse is the security guards are also afraid to go with the ambulance team.”****KII-MCA 1****.*

In addition, insecurity hinders attraction of skilled health professionals from other areas outside Garissa as alluded by some study participants. Yet, efforts to change the narrative of insecurity is masked by other people’s perceptions of insecurity in the area which depicts the community as hostile.

*“Security is one, most experts do not want to hear about Garissa or Mandera or Wajir, that is why we miss on specialized skills because they don’t want to come to this part of the country.”****KII-MCA 2***

Recognising the enormity of the security issue and the disincentive it creates, especially for service providers who come from other parts of Kenya, the Garissa County government has initiated measures of hiring skilled individuals from the area who do not stand out as targets and less threatened and affected by insecurity.

*“Last year a lot of health workers working in the rural health facilities in far and hard to reach areas, were withdrawn because of insecurity and most of health facilities closed for quite a long time. So to address this, the County has started recruiting locals who are not being threatened by security Issues-Somalis for that matter unlike other Kenyans from other Counties who are prone to security risks”****KII-MCA 2***

*“The government and the security agency should be involved in provision of security to healthcare workers.”****KII-HM 2***

### Service side barriers

Availability of care, human resource capacity and infrastructure were identified as service side barriers.

#### Availability of care

It was reported that some health facilities do not operate at night. Even those that open at night, the level of available staff is low to adequately attend to all those in need. Some of the health facilities lack essential drugs and patients are forced to buy drugs from private outlets.

*“We are not on night duties due to shortage of staff and insecurity at night. The services are only limited to day time”****KII-HM 1***

Often, unavailability of drugs caused users to lose trust in healthcare providers and centres when they do not receive all the services they require. Though healthcare providers identified it as a wider supplying system issue rather than an in a facility level one.

*“…mothers and children don’t get what they came for here. Medicine is one of it, mothers are prescribed medication for and asked to buy from the pharmacies.”****FGD CHVs***

*“For medication stock out, maybe when the drugs are out of stock we have to order them and may there is delay of supply because sometimes there is a delay in the supply. For vaccination we don’t have stock out because before the vaccines are over we have to order and we get them...”****KII HM-1***

#### Human resources capacity

Inadequate human resource affects implementation of MNCH services. The number of healthcare workers offering MNCH services are deemed inadequate to serve the number of pregnant mothers seeking services. Fewer staff creates the impression that the quality of care is poor, including long waiting times. As supported by the excerpts below:

*“We have staff shortage and those we have are working long hours to provide services to all mothers visiting the health facility. This is a fact that we cannot keep ignoring.”****KII-HM 2***

Some patients have adopted unorthodox ways in order to receive services quicker. A practice that respondents were not happy about as they are not treated equally. The number of staff available also influenced the times that women access care which was mostly during the day. This was because staff were not always available at night or on weekends. The issue was attributed to the lack of enough health workers to provide the care needed at all times.

*“I came here on Sunday night and there was one nurse and the watchman only. Then the nurse referred me to General hospital****.” FGD Women (15- 29 years)-SIMAHO***

*“…we have staff that are working through the hours, it’s the fact that we cannot ignore we have shortages in our staff, as much as we want to provide services in each or close to all our mothers.”****KII HM3***

Inadequate human resource also greatly increases the workload of staff. This is particularly true for female healthcare workers who are fewer than their male counter parts. Yet, women prefer to be attended to by them.

*“Some employees over work especially the female staff because the clients want to be attend to by female staff especially in the maternity and immunization. The female staffs are overworking”****KII-HM 2***

There are suggestions to increase the number of staff to reduce the waiting time and also should be available to offer services at night.

*“…staff numbers should be increased to serve the mothers in order to reduce the long queue. We request they employ adequate staff for example nurses and midwives that can attend to us at night.”****FGD women (15-29 years)-Iftin****.*

#### Infrastructure

Infrastructure was mentioned to be an important barrier to why women didn’t access MNCH care in health facilities. Many participants reported that health facilities lacked the space and capacity to provide quality care to pregnant women, postpartum women and their newborns. They particularly reported the inadequate beds and lack of water in the health facilities.

*“… We need extra rooms for the hospital since they are no spaces left for new equipment to be stored in. We also need beds and mattresses because one time a mother came to give birth at the hospital and she found there were no remaining beds. We took mattress from the male ward and was laid on it. We seriously need more rooms because the people of this area are increasing and there are no spaces in the ward. Even the staffs working there don’t have that privacy to change clothes. Even there is no waiting bay.”****FGD Women (30+ years) –Iftin***

The problem was further aggravated by the lack of theatres for services such as surgery which is a key requirement for women who developed complications during delivery and require a caesarean section. Together with the cases of newborns who experienced complications, women had to be referred to the County Referral hospital which delays timely care.

“*We would like to have a theatre just in case of complications or emergencies. We have doctors here but not the equipment, referring one to PGH takes long time because of ambulance and arrangements.****FGD women (15-29 years) -Iftin***

Respondents voiced their frustrations over the poor infrastructure including lack of basic amenities of some facilities which has been a constant battle with those in positions of leadership.

*“We would like the county government to build a theatre for the hospital. Clearly we are lacking theatres and lab for blood bank. We need extra rooms for the hospital since they are no spaces left for new equipment to be kept in. We also need beds and mattresses because one time a mother came to give birth at the hospital and she found there were no empty beds.”****FGD women (30+ years)-Iftin****.*

## Discussion

This study provides results that are part of the impact evaluation of a package of interventions aimed at increasing uptake of maternal, new-born and child health services in Garissa County. A similar assessment was done at baseline and the interpretation of findings here intimately draw on observations from the baseline assessment [21]. We found generally positive attitudes towards MNCH care services. However, from the findings, it is clear that some of the barriers identified in the pre-intervention period are enduring as they were mentioned as key barriers to accessing and using care at the end of the intervention. Generally, though not quantifiable in this study, there seems to be a shift towards reporting more structural issues/barriers as opposed to individual and household-level factors/barriers.

Lack of emergency transport, insecurity, and long distances to health facilities were the predominant barriers mentioned. Studies have demonstrated that the distance to health facilities reduces utilization of maternity services [24–27]. Limited transportation options play a crucial role whether or not pregnant mother can reach health facility in good time [28–35]. This is especially important if the need for transport comes at night times as it comes with a higher cost [33–38]. These barriers are systemic, complex and are closely tied to the general socio-economic development of the County and the region. The region has been marginalized for decades in terms of social development as well as a high level of insecurity [39]. This has plagued the wider north-eastern region for years as it is characterized by high volatility manifested in periodic outburst of inter-communal violence and attacks from terrorist groups like Al-Shabaab [40]. Ethnographic and qualitative studies have linked conflict to reduced utilization of healthcare through destruction of health facilities, intimidation of medical personnel and creating atmosphere that discourages travel [41–44]. Recent Al-Shabaab attacks in Garissa County has resulted in many non-locals health workers refusing to return to the area leaving behind large gaps in the health sector. Conflict is also a major determinant of poverty through forms of unemployment and inequality which have effect on economic structures [45, 46]. With the devolved governance system coming into effect, things are slowly changing but a lot needs to be done to bring Garissa to a comparable level with other counties, for example those in central Kenya.

The other dominant class of barriers mentioned relate to the unavailability and readiness of health services provision. There is a general uneasiness among women, men, religious and cultural leaders on the issue of male maternity service providers. This issue was identified at the baseline survey [21] and communicated to the health services management. The health services management, through affirmative action, have prioritized hiring of female service providers for maternity units. However, given the significantly high financial resource implications, it might take a long time to fully meet this aspiration. In the meantime, with continued implementation of the other components of culturally acceptable and sensitive service package which was provide under the project intervention, this barrier is likely to gradually become less of a hindrance to access and utilisation. Studies elsewhere have shown that cultural competence and respectful maternity services improves service utilization [47–49]. This happens because providers are aware and likely to provide options that clients are comfortable with without judging them. On the other hand, there is continued realization by many community members that the issue at hand is more cultural than religious, and as such not a taboo as reflected in some of the qualitative interviews.

While individual and household-level barriers were not rated highly as barriers at end of the project, we anticipate that some of the issues identified will remain challenges for some time. These include poverty, lack of information, the dislike of male service providers, as well mistrust of service providers. Social changes occur overtime and often have profound and long term consequences [50].

Our study contributes to the growing literature on persistent barriers to access and utilization of MNCH services in culturally dominated and less endowed setting. The persistent barriers identified are interdependent and tackling them requires a holistic approach. Benefits of integrating culturally appropriate services in MNCH can be achieved if all stakeholders are involved. Understanding the importance of local communities in health will be useful to public health intervention in less endowed areas. The intervention improved the link between service providers, CHVs and TBAs. Policy initiatives to address current structural bottlenecks would provide away forward towards achieving better MNCH indicators in the County. To further improve the access and utilization of MNCH services, Garissa County must ensure health facilities in their jurisdiction maintain and offer culturally appropriate and sensitive services.

### Strengths and limitations

All authors a background in health and some are involved in maternal care. For a qualitative study, it is extensive with 125 participants who came from the three sites, different age groups, different gender and different levels of exposure to maternal care. This is part of a larger study project from which one qualitative study has been published. This enabled the interviewers to triangulate the themes from this study with those found in the pre-implementation study, as well as those existing in literature. This heightened the validity of the results of the study.

This intervention period was relatively short for social change. The short intervention period was dictated by the limited funding available for the intervention. Self-reported experiences and believes may not reflect action so we cannot with certainty assume that the intervention lessened or removed some of the barriers as some of the answers might be given just for the sake of being socially desirable. Also the interviews were not conducted with the same people at baseline and end-line and as such, the observed differences might be a random variation in opinion among people interviewed and not related to intervention.

## Conclusion

Given the observation that individual, household and community level barriers seem to be less problematic, we can attribute this apparent change to the effect of the intervention and as such we recommend the sustenance of the key intervention components even on a scaled down level. The grass root service providers (CHVs) need to be supported to continue with health education, and provision of culturally acceptable and sensitive service at health facilities need to be mainstreamed in both private and public facilities. These interventions are catered for in the current health policy and community strategy and strategic plans and they do not require much extra policy intervention to actualize.

## Data Availability

All data will be made public and available after 2 years on the APHRC Microdata portal: http://microdataportal.aphrc.org/index.php/catalog. Contact Abdhalah Kasiira Ziraba; email: aziraba@aphrc.org to request access to data and materials.

## Abbreviations

ANC: Antenatal Care
CASS: Cultural Appropriate and Sensitive Services
CHVs: Community Health Volunteers
CICF: County Innovation Challenge Fund
FANC: Focused Antenatal Care
GK: Government of Kenya
MCA: Member of County Assembly
MNCH: Maternal Newborn and Child Health
MPDSR: Maternal and Perinatal Death Surveillance and Response
RMNCH: Reproductive, Maternal, Newborn and Child Health
PNC: Post Natal Care
SDGs: Sustainable Development Goals
TBAs: Traditional Birth Attendants
